# Types of species of Apionidae (Coleoptera) described by Carl Peter Thunberg (1743–1828) with description of a new genus

**DOI:** 10.3897/zookeys.317.5477

**Published:** 2013-06-22

**Authors:** Miguel A. Alonso-Zarazaga

**Affiliations:** 1 Depto. de Biodiversidad y Biología Evolutiva, Museo Nacional de Ciencias Naturales (CSIC), José Gutiérrez Abascal, 2, E-28006 Madrid, Spain

**Keywords:** Weevils, *Thunbergapion*, *Apion*, Carl Peter Thunberg, new genus, new combination, new synonymies, morphology, systematics, key

## Abstract

The type specimens of species of Apionidae described by Carl Peter Thunberg are reviewed and lecto- and paralectotypes are designated for *Apion craccae* Thunberg, 1813, *Apion limbatum* Thunberg, 1813, *Apion punctigerum* Thunberg, 1815 and *Apion astragali* Thunberg, 1815. A new genus *Thunbergapion* (type species *Apion limbatum* Thunberg, 1813) is described, figured and placed in the tribe Aplemonini Kissinger, 1968. The new combination *Thunbergapion limbatum* (Thunberg, 1813) is proposed. A key to the known South African genera of the tribe is given. The following new synonymies are established: *Oxystoma craccae* (Linnaeus, 1767) = *Apion craccae* Thunberg, 1813 **syn. n.**, *Ischnopterapion (Ischnopterapion) loti* (Kirby, 1808) = *Apion punctigerum* Thunberg, 1815, **syn. n.**, and *Pseudoprotapion astragali* (Paykull, 1800) = *Apion astragali* Thunberg, 1815, **syn. n.**

## Introduction

Carl Peter Thunberg (*1743, †1828) was a Swedish botanist and entomologist and disciple of Carolus Linnaeus. He stayed in South Africa from 16 April 1772 to 2 March 1775, when he sailed to Batavia. During his stay in the Cape Colony of the Dutch East India Company (now South Africa) he undertook several short excursions and three long trips beyond the hinterland to collect animals and plants. He visited the colony again during his return journey to Europe, from 27 April to 15 May 1778 (Forbes 1986; Muller and Rookmaaker 1992).

As a result of his trips, Thunberg (1813) presented a list of hitherto known weevil species from the Cape of Good Hope area. He added short diagnoses and references to those previously described. The genera and species treated are summarised in Table 1. None of the new species have been treated in later regional or worldwide catalogues, although they were included in Sherborn’s *Index Animalium*. The new species were:

*Apion craccae* (p. 382)

*Apion limbatum* (p. 382)

*Curculio papillaris* (p. 384)

*Curculio gibbosus* (p. 388) (non Gmelin, 1790, nec Paykull, 1792)

*Curculio analis* (p. 389) (non Olivier, 1790)

*Curculio felinus* (p. 390)

*Curculio cyaneus* (p. 390) (non Linnaeus, 1758, nec Herbst, 1795, nec Weber, 1801)

*Curculio elongatus* (p. 390) (non Fabricius, 1775, nec Goeze, 1777, nec Paykull, 1792)

*Curculio cinctus* (p. 391) (non Drury, 1782, nec Geoffroy, 1785, nec Rossi, 1790, nec Paykull, 1792, nec Olivier, 1807)

*Curculio octoguttatus* (p. 391)

*Curculio zebrae* (p. 392)

*Curculio margaritaceus* (p. 393).

**Table 1.** Genera and number of species treated in Thunberg (1813), and new species described therein. Names as given originally.

**Genus**	**Total species**	**New species**
*Cordyle*	2	0
*Lixus*	4	0
*Rhynchaenus*	12	0
*Apion*	2	2
*Curculio*	32	10
*Brachycerus*	32	0
*Platyrynchos*	1	0
*Attelabus*	4	0
*Rhinomacer*	1	0

The descriptions of these species are insufficient to allow their proper placement to genus. Those described under *Apion* will be treated below, after studying their type specimens. Those described under *Curculio* may merit a separate study, although it appears from the descriptions that probably the first four and the last two may belong to the Cyclominae (Cyclomini, Hipporhinini). As can be seen from the list above, five of these available names are potentially valid but five are permanently invalid because of primary homonymy.

Thunberg (1815) published another revision of the genera and species of weevils known to him (including Bruchinae), giving descriptions for all of the genera (new were *Amblycerus*, *Chyphus*, *Platyrynchus* and *Temnocerus*), enumerating the species belonging to each genus and describing, apparently as new, 29 species. Of these, three were new species attributed to the genus *Apion*, namely *Apion sanguineum*, *Apion punctigerum* and *Apion astragali*. Contrary to the species included in his previous article, these have usually been included in the catalogues of Coleoptera and are well known, at least by name. A summary of the genera and species treated is included in Table 2.

**Table 2.** Genera and number of species treated in Thunberg (1815), and new species described therein. Names as given originally.

**Genus**	**Total species**	**New species**
*Cordyle*	12	3
*Ramphus*	12	1
*Lixus*	13	2
*Rynchaenus*	116	8
*Rynchites*	12	3
*Apion*	12	3
*Brentus*	4	0
*Curculio*	99	4
*Cossonus*	2	0
*Hyselinus*	14	0
*Antribus*	5	0
*Amblycerus*	8	3
*Platyrhynchus*	2	1
*Temnocerus*	2	0
*Brachycerus*	29	0
*Chyphus*	1	0
*Attelabus*	3	1
*Bruchus*	12	0
*Rhinomacer*	2	0

## Material and methods

Type specimens of Thunberg’s collection were received from the Zoological Museum of Uppsala University (Sweden). The specimens were studied under a binocular Leica Wild MZ8 microscope and photographed with an Olympus C7070WZ camera mounted on the same microscope or on a photographic frame Kaiser RA1. Microscope slides were studied and photographed with the same camera mounted on a Leitz Diaplan microscope, and some details were drawn by using a drawing tube. Extended focus images were generated using the software CombineZP. The programs Adobe Illustrator CS5.0 and Adobe Photoshop CS5.0 were used for image postproduction and mounting.

Dissection methods and nomenclature of genitalia follow Alonso-Zarazaga (1990). Genitalia and terminalia have been placed in a drop of DMHF on an acetate card accompanying the specimen for long term conservation (Steedman 1958; Bameul 1990).

Labels are described as they were copied by the curators, since they were retained in the drawers. These labels are red and read “Uppsala Univ. Zool. Mus. / Thunbergsaml. nr. # / *Genus species* / *##* TYP.” In the treatment for each species, only the data represented by the marks # and ## are given. Specimens designated as lectotypes are labelled as such, with white labels bordered and printed in red: (PARA)LECTOTYPUS / Genus / species / Thunberg, 181(3-5) / Alonso-Zarazaga des. 2013. Each specimen was also provided with white identification labels printed in black.

## Taxonomy

### Apion craccae

Thunberg, 1813: 382

http://species-id.net/wiki/Apion_craccae

#### Original description.

*A. craccae*: nigrum opacum, elytris striatis. *.

*Magnitudine* pulicis, globosum, nigrum, opacum, omnino simile A. craccae, europaeo.

#### Material studied.

I have received four specimens from the collection Thunberg: 539, Mus.Thunb. Pierced midway with a long thin pin without head, abdomen glued to a white card below.

1188, Cap. Pierced low with a short thick pin with head.

1189, Sv. Pierced midway with a long thin pin with head. The abbreviation “Sv.” stands for “Svecia” [= Sweden].

15075, no data. Pierced midway with a long thin pin with head.

The specimen with the number 1188 has a further label reading “Cap” and is here designated as lectotype and labelled accordingly. Although the specimens with the numbers 539, 1189 and 15075 are pinned in a similar manner, they cannot be considered as syntypes as their label data do not conform with the original locality, Cape Province, as it can be inferred from the title of the article. However, all four specimens belong to the same species, the European *Oxystoma craccae* (Linnaeus, 1767). *Apion craccae* Thunberg, 1813 is thus a new synonym of the name of Linnaeus’ species. It is almost certain that the locality on the lectotype is a mistake.

### Apion limbatum

Thunberg, 1813: 382

http://species-id.net/wiki/Apion_limbatum

Figs 1–2 , 6 –11

#### Original description.

*A. limbatum*: cinereo-fuscum, elytrorum sutura basi margineque externo albidis.

*Magnitudo* minoris pediculi; totum glabrum, cinereo-coerulescens.

*Elytra* striata sutura basi margineque externo albo.

*Pedes* inermes.

#### Material studied.

One male specimen, glued to a pin 20.4 mm long and 0.59 mm in diameter with the following data: 1293, Cap. The original Thunberg labels, which I have not seen, read: “*Apion*” (genus label) and “*limbatum*. Cap.” (species label). It is here designated as the lectotype of this species. The specimen is severely damaged, the abdomen is broken and separated from the body and the elytra are open, but fortunately all legs and antennae are entire. I have been able to recover the abdomen and prepare the remainder of the ventrites, terminalia and genitalia in a drop of DMHF (water- or ethanol-soluble resin) on an acetate card. Study of the specimen showed it to be a member of an hitherto undescribed genus, which is described below.

**Figures 1–5.**
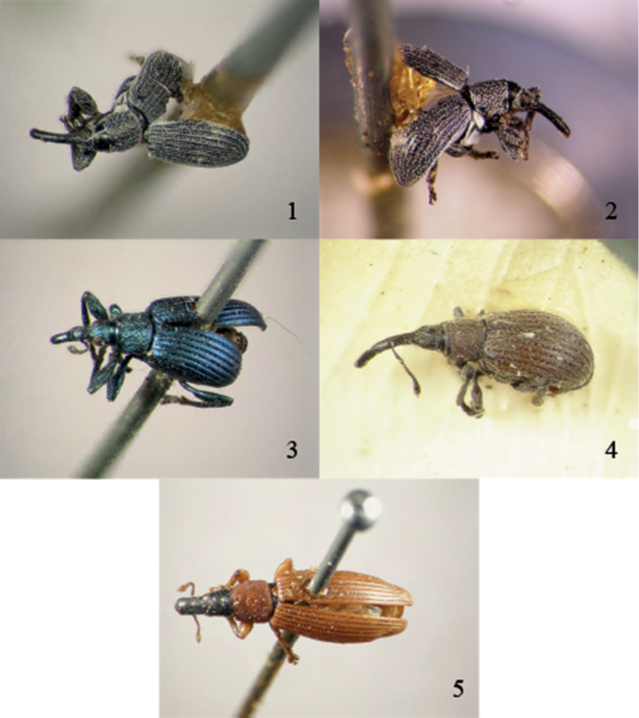
Type specimens in Thunberg’s collection **1** *Apion limbatum* Thunberg, 1813, lectotype male, dorsal view **2** *Apion limbatum* Thunberg, 1813, lectotype male, lateral view **3** *Apion astragali* Thunberg, 1815, lectotype male, dorsolateral view **4** *Apion punctigerum* Thunberg, 1815, lectotype, dorsolateral view **5** *Apion sanguineum* Thunberg, 1815, syntype, dorsal view.

**Figures 6–8.**
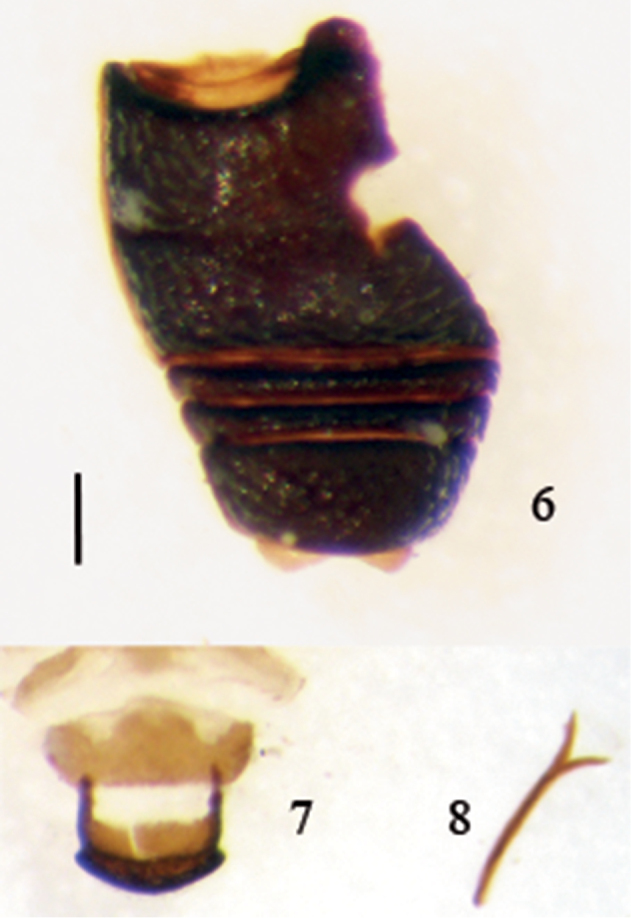
*Thunbergapion limbatum* (Thunberg, 1813) **6** Abdominal sternites, ventral view **7** Male pygidium, dorsal view **8** Male ninth sternite (spiculum gastrale). Scale: 100 µm.

**Figures 9–11.**
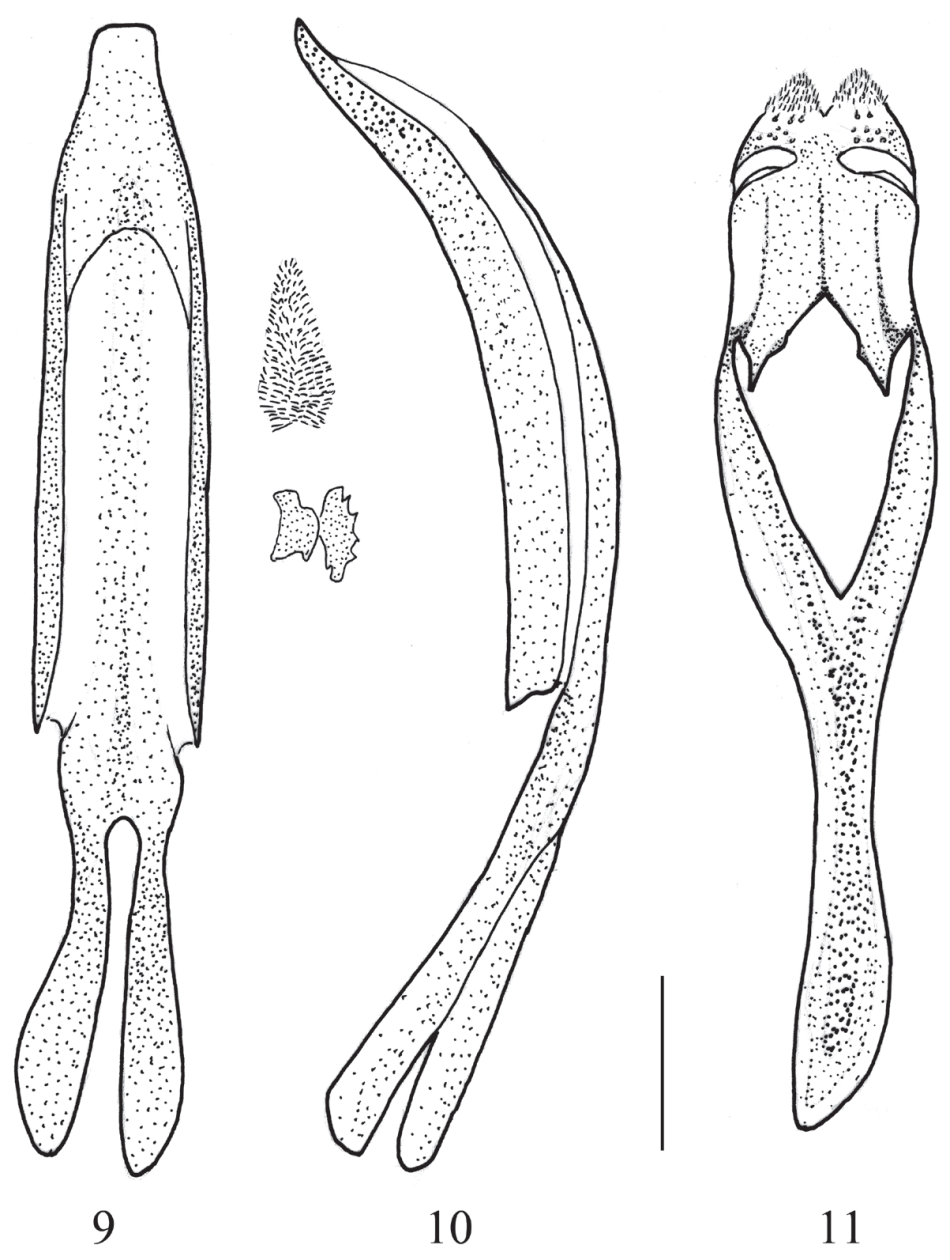
*Thunbergapion limbatum* (Thunberg, 1813) **9** Penis, dorsal view, inner sac armature figured outside **10** Penis, lateral view **11** Tegmen, dorsal view. Scale: 100 µm.

### Apion sanguineum

Thunberg, 1815: 118

Fig. 5

#### Note.

This species is represented by a single pinned specimen belonging, as usually accepted, to the genus *Tanaos*. I refrain from redescribing it here, since a revision of *Tanaos* is in preparation by my good colleague and friend Dr. Marek Wanat (Wrocław, Poland).

### Apion punctigerum

Thunberg, 1815: 118

http://species-id.net/wiki/Apion_punctigerum

Fig. 4

#### Original description.

*A. punctigerum*: niger, elytris nigris, striatis.

*Habitat* in Smolandia Sveciae.

*Magnitudine* pediculi totus opacus, niger.

*Elytra* gibba, striata, atra.

#### Material studied.

I have received three specimens, as follows: one divided into three parts and glued onto a square white card, this on a short thin pin with head, with data: 543a, Mus. Thunb.; one headless, worn, pinned high on a long thin pin with head, with data: 543b, Mus. Thunb.; and a third glued to a rectangular label, this on a short thick pin with head, with data: 1191, Smol. The first specimen is a female of *Aspidapion radiolus* (Marsham, 1802), the second a female of either *Ceratapion gibbirostre* (Gyllenhal, 1813) or *Curculio carduorum* (Kirby, 1808), and the third is an etiolated (probably female) specimen of *Ischnopterapion (Ischnopterapion) loti* (Kirby, 1808). The first two are coloured brightly, with the elytra more or less metallic, and do not fit Thunberg’s description. The third fits Thunberg’s description, although the body colour has become brownish, and is labelled with the type locality, Smolandia (= Småland, Sweden). I hereby designate this specimen as the lectotype of *Apion punctigerum* Thunberg, 1815, and its name becomes a new synonym of that of *Ischnopterapion loti*.

### Apion astragali

Thunberg, 1815: 118

http://species-id.net/wiki/Apion_astragali

Fig. 3

#### Original description.

*A. Astragali*: caerulescens nitidus, elytris striatis.

*Habitat* in Vestrogothia Sveciae.

*Totus* supra nitens, caerulescens; subtus magis niger magnitudine pediculi.

*Elytra* gibba, striata.

#### Material studied.

I have received three specimens with red type labels as above, carrying the numbers 1206, 1289 and 1290. Only specimen nr. 1289 has an additional label reading “Vestrog.”. This specimen, pinned high on a long pin with head, is a female *Pseudoprotapion astragali* (Paykull, 1800) and is here designated as lectotype. This allows establishment of the following new synonymy: *Pseudoprotapion astragali* (Paykull, 1800) = *Apion astragali* Thunberg, 1815. Specimen 1206 is a male of the same species and is designated as paralectotype. However, specimen 1290 does not fit the original description and is not considered to represent a syntype, not being bright bluish as the others but dull bronze-black. It is a female of *Aspidapion radiolus* (Marsham, 1802). Thunberg’s original species label of this specimen carries a question mark and the letter β, in contrast with the other two, which carry the letter α.

### Thunbergapion

Alonso-Zarazaga gen. n.

urn:lsid:zoobank.org:act:EAAA82B8-0D85-462D-8659-E2F4A1D2FA47

http://species-id.net/wiki/Thunbergapion

Figs 1–2 , 6 –11

#### Type species.

*Apion limbatum* Thunberg, 1813. Gender neuter.

#### Description.

With the characters of tribe Aplemonini Kissinger, 1968, as detailed by Alonso-Zarazaga (1990: 88).

*Integument*. Colour black, elytra with a faint leaden-bluish glint.

*Vestiture* subsquamose, scales elliptical to lanceolate, in one ordered row on each interstria, sometimes row disordered in the middle of some interstriae. Pronotal vestiture centripetal.

*Rostrum* 1.28 × as long as pronotum, sparsely squamose in basal third, almost glabrous in apical two thirds, in dorsal view sides slightly convergent to mesorostrum, tubiform from mesorostrum to apex, 5.2 × as long as wide at apex, mesorostrum not dilated, punctures dense and large at base, becoming progressively less dense and smaller towards apex, integument scarcely microreticulate, bright; in side view straight at basal half, weakly deflexed and curved in apical half. Scrobes weakly sulciform.

*Head* short, separated from occiput by a dorsal transversal depression, forehead little narrower than rostral apex, with two irregular lines of scale-bearing punctures near inner margin of eyes and a central oblong-elliptical, moderately deep fovea. Eyes slightly oblong, moderately convex. Temples very short, ca. ¼ length of eye.

*Antennae* inserted at basal 0.33 of rostrum. Scapes moderately long, 1.33 × as long as mesorostral width. Clubs elongate, fusiform, 2.6 × as long as wide, sutures visible.

*Pronotum* moderately transverse, 0.90 × as long as wide, maximum width near middle, weakly narrowed towards base, more strongly so towards apex. Basal flange absent. Prescutellar fovea obsolete, reduced to a very weak oblong depression near base, not longer than a puncture. Base straight.

*Scutellum* small, subtriangular, glabrous, impunctate.

*Elytra* oval-elongate, ca. 1.6 × as long as wide (a more precise measure impossible due to the condition of the specimen), widest at middle, humeral calli moderately developed. Striae at apex joining 1+2+9, 3+4, 5+6, 7+8, 2^nd^ not extended outwards, at base 1^st^ shortened before scutellum. No specialized setae.

*Ventral areas*. Mesocoxae tangent. Median apophysis of mesoventrite triangular, short, median apophysis of metaventrite rather narrow and elongate, tuberculiform. Anterior metasternal rim fine. Abdomen (Fig. 6) strongly and densely punctate, punctures moderately deep, scales not condensed, finer than on disc of metaventrite. First abdominal ventrite ca. 1.8 × as long as 2^nd^, this as long as 5^th^. Suture I marked. Fifth ventrite subtruncate at apex.

*Pygidium* (Fig. 7) of apionine type, with apical flange weakly and widely notched medially.

*Legs*. Tibiae unarmed. Tarsi robust, 1^st^ protarsomere 1.11 × as long as wide, 2^nd^ transverse, 0.73 × as long as wide, 0.8 × as long as 1^st^, 3^rd^ 0.69 × as long as wide, deeply bilobed, onychium 3.24 × as long as wide, surpassing lobes of 3^rd^ tarsomere by 0.54 × its own length. No tarsomere ventrally spined. Claws weakly incrassate at base, not toothed.

*Genitalia and terminalia*: Tegmen (Fig. 11) with parameroid lobes shorter than dorsal portion of ring, apically subtriangular, with short apical membranous microsetose area, basal sclerotized area with some irregularly arranged short macrochaetae and sensilla. Fenestrae separated, curved. Prostegium fused to free ring, with a deep triangular notch and one basal tooth on each side, the teeth with a small inner prominence. Linea arquata obsolete. Free ring and manubrium flattened. Penis (Figs 9–10) weakly compressed, apex of pedon prominent as a trapezoidal truncate plate in dorsal view, in side view gently curved with the apex weakly recurved. Internal sac with apical spicules uniform, ca. 10 µm long, rather condensed in a subtriangular patch, medially with two pieces apparently formed by conglomerate teeth, ca. 80 µm long, and basally with some sparse minute spicules and asperities. Spiculum gastrale (Fig. 8) Y-shaped, slightly asymmetrically curved, manubrium ca. 2.5 × as long as arms.

#### Note.

This description is based only on the male sex, the female is presently unknown.

#### Material examined.

The lectotype male of *Apion limbatum* Thunberg, 1813, as mentioned above.

#### Etymology.

This genus is named after Carl Peter Thunberg, the illustrious Swedish naturalist and almost certainly the collector of the type species, during his travels in present-day South Africa.

### Thunbergapion limbatum

(Thunberg, 1813) comb. n.

http://species-id.net/wiki/Thunbergapion_limbatum

#### Description of lectotype.

*Measurements* (in µm): Body length (not including head and rostrum): 1834. Rostrum: length: 628; width (apical): 120, (mesorostral): 126. Distance from antennal insertions to base: 209. Forehead: width: 107. Eyes: length: 168. Scapes: length: 168; maximum width: 26. Desmomeres 1–7 (length × width): 52 × 26, 31 × 21, 31 × 21, 31 × 21, 31 × 21, 21 × 21, 21 × 21. Clubs (length × width): 178 × 68. Pronotum: length: 492; width (apical): 429, (maximal): 544, (basal): 492. Elytra: length: 1342; joint width (at humeri): 650, (maximal): 900 (last two gross estimations due to open elytra).

*Vestiture* of upperside consisting of short, lanceolate to elliptical white scales, ca. 30 ×10 µm on pronotum, sparse, not or hardly surpassing margins of punctures, those on elytral interstriae similar, usually touching one another in the row, rarely overlapping, forming duplicate irregular rows in some parts along the interstriae, those of striae smaller, about half the size of the interstrial ones, not touching; elytral scales condensed on the basal third of scutellar interstriae and on basal two thirds of the costal interstriae; ventral scales finer and longer (ca. 50 ×7 µm), condensed on mesoventrite and mesopleural sclerites and on sides of metaventrite and metanepisternum, except the strikingly bare anterolateral angles of metaventrite, sparser on metasternal disc.

*Antennae* dark piceous brown, shortly setose. Funicles subcylindrical, rather compact.

*Pronotum* with large superficial punctures ca. 30 µm in diameter, separated by ca. 10 µm, integument weakly microreticulate, shining.

*Elytra* with striae about half as wide as interstriae.

*Wings*. Macropterous.

*Legs* robust, profemora 2.34 × as long as wide, protibiae straight, widening towards apex, 5.35 × as long as wide.

## Discussion

The new genus *Thunbergapion* belongs to the tribe Aplemonini Kissinger, 1968 by the presence of a male pygidium of the normal apionine type, the abdominal ventrites similarly punctate, the manubrium of the spiculum gastrale much longer than the arms, the absence of a specialized seta on the middle of the 7^th^ interstriae, the deeply notched prostegium with one tooth on each side, fused to the free ring, the elytral striae 1, 2 and 9 not more impressed at apex than on disc, and the tangent mesocoxae. The simple claws and the almost straight rostrum are also common features in this tribe.

Peculiar characters of this genus are the squamose vestiture, condensed on the sutural and marginal interstriae and on the meso- and metapleurites, the long, almost straight rostrum, the fusiform antennal clubs and the two strongly dentate median pieces of the internal sac.

This genus seems to include also at least two other undescribed species from the same area (Marek Wanat, pers. comm.) and may constitute and endemic element.

Using the key to the genera of the Palaearctic region (Alonso-Zarazaga 1990, p. 88 and ff.), which includes all the know genera of Aplemonini except the Mexican *Femtapion* Kissinger, 1991, doubtfully a member (Alonso-Zarazaga and Wanat in press), *Thunbergapion* agrees with the characters of couplet 3 and runs to either *Pseudoperapion* Wagner, 1930 or *Pseudostenapion* Wagner, 1930. It differs from both these genera by the squamose vestiture (piliform in the others), the longer rostrum (shorter than pronotum in the others), the narrower forehead compared with the rostral apex (much wider than apex in the others), the scapes longer than the mesorostral width (0.5–0.7 × as long in the others), the longer, fusiform clubs (ca. 1.9 × as long as wide and oboval in the others), the male pygidium with rim margin weakly notched (angulate in the others), the obsolete linea arquata of tegmen (well marked in the others) and the two medial toothed structures of the internal sac (only small teeth, spicules or asperities in the others).

*Thunbergapion* additionally differs from *Pseudostenapion* by its shorter, plumper form (elongate in *Pseudostenapion*), the moderately shiny integument (matt in *Pseudostenapion*), the punctate prorostrum (smooth and shining in *Pseudostenapion*) and the transverse pronotum (oblong in *Pseudostenapion*); the tegmen in both genera is very similar in general and the penis shows a prominent apical knob in *Pseudostenapion*.

From *Pseudoperapion* *Thunbergapion* additionally differs by its longer 2^nd^ abdominal ventrite (very short in *Pseudoperapion*), the transverse 2^nd^ tarsomere (isodiametric in *Pseudoperapion*) and the shorter manubrium (ca. 7 × as long as arms in *Pseudoperapion*); the apex of the penis is slightly recurved in both genera, but its general shape is rather cylindrical and elongate in *Pseudoperapion*. *Thunbergapion* also shares with *Pseudoperapion* the condensed vestiture in some ventral areas.

Because of its elongate rostrum, *Thunbergapion* shows some similarity also to *Cistapion*, a Mediterranean endemite and probably a relict. The genital structures are very similar in both genera, except for the presence in *Cistapion* of long macrochaetae on the parameroid lobes of the tegmen and a well marked linea arquata. In addition, the temones of the penis are very short in this genus (less than half the length of the pedon), which is not the case in *Thunbergapion*.

The species of *Pseudostenapion* and *Pseudoperapion* are stenophagous on species of the plant genus *Hypericum* L. (Hypericaceae), and it is possible that *Thunbergapion limbatum* also lives on plants of this genus (there are six species in the Cape Province).

In South Africa, three genera of the tribe Aplemonini are present: *Aplemonus* Schoenherr, 1847, *Perapion* Wagner, 1907 and *Thunbergapion* gen. n. These genera can be distinguished using the following simple key:

**Table:** 

1	Claws toothed. Elytra strongly convex behind middle, anteriorly concave in side view. Host: *Zizyphus* spp. (Rhamnaceae)	*Aplemonus*
–	Claws simple or slightly incrassate at base. Elytra uniformly and weakly convex in side view	2
2	Antennal clubs oboval. Metasternal apophysis shortly triangular. Antennal scapes at most as long as mesorostral width. Vestiture piliform. Host: *Emex* spp. (Polygonaceae)	*Perapion*
–	Antennal clubs fusiform. Metasternal apophysis narrow and elongate, tuberculiform. Antennal scapes 1.33 × as long as mesorostral width. Vestiture squamose. Host unknown	*Thunbergapion*

## Supplementary Material

XML Treatment for Apion craccae

XML Treatment for Apion limbatum

XML Treatment for Apion sanguineum

XML Treatment for Apion punctigerum

XML Treatment for Apion astragali

XML Treatment for Thunbergapion

XML Treatment for Thunbergapion limbatum

## References

[B1] Alonso-Zarazaga MA (1990) Revision of the supraspecific taxa in the Palaearctic Apionidae Schoenherr, 1823 (Coleoptera, Curculionoidea). 2. Subfamily Apioninae Schoenherr, 1823: introduction, keys and descriptions. Graellsia 46: 19-156

[B2] Alonso-Zarazaga MA Wanat M (in press) Brentidae Apioninae. In: Leschen RAB, Beutel RG (Eds) Handbook of Zoology/ Handbuch der Zoologie, Band 4: Arthropoda, 2. Hälfte: Insecta, Coleoptera, Beetles, Volume 3: Morphology and Systematics (Phytophaga). De Gruyter, Berlin, Boston.

[B3] Bameul F (1990) Le DMHF: un excellent milieu de montage en entomologie. L’Entomologiste (Paris) 46(5) 233–239

[B4] Forbes VS (Ed) (1986) Carl Peter Thunberg. Travels at the Cape of Good Hope 1772–1775. The Van Riebeeck Society, 2^nd^ Series No. 17, Cape Town, 366 pp.

[B5] Muller S Rookmaaker LC (1992) The South African insects described by Carl Peter Thunberg (1743–1828). Journal Namibia Scientific Society 43: 81-90

[B6] Steedman HF (1958) Dimethyl Hydantoin Formaldehyde: a new water-soluble resin for use as a mounting medium. Quarterly Journal of Microscopical Science 99 (4): 451-452

[B7] Thunberg CP (1813) Coleoptera Rostrata Capensia. Mémoires de l’Académie de Saint Petersburg 4: 376-400

[B8] Thunberg CP (1815) De Coleopteris Rostratis commentatio. Nova Acta Regiae Societatis Scientiarum Upsaliensis 7: 104-125

